# Effective biofilm eradication in MRSA isolates with aminoglycoside-modifying enzyme genes using high-concentration and prolonged gentamicin treatment

**DOI:** 10.1128/spectrum.00647-24

**Published:** 2024-08-27

**Authors:** Kohei Ando, Satoshi Miyahara, Shuhei Hanada, Kazumasa Fukuda, Mitsumasa Saito, Akinori Sakai, Akihiro Maruo, Yukichi Zenke

**Affiliations:** 1 Department of Emergency and Intensive Care Medicine, School of Medicine, University of Occupational and Environmental Health, Kitakyushu, Japan; 2 Department of Microbiology, School of Medicine, University of Occupational and Environmental Health, Kitakyushu, Japan; 3 Department of Orthopedic Surgery, School of Medicine, University of Occupational and Environmental Health, Kitakyushu, Japan; 4 Department of Orthopedic Surgery, Hyogo Prefectural Harima–Himeji General Medical Center, Himeji, Japan; University of Torino, Turin, Italy

**Keywords:** MRSA, biofilm, aminoglycoside-modifying enzyme genes, gentamicin, minimal biofilm eradication concentration (MBEC)

## Abstract

Bone and soft tissue infections caused by biofilm-forming bacteria, such as methicillin-resistant *Staphylococcus aureus* (MRSA), remain a significant clinical challenge. While the control of local infection is necessary, systemic treatment is also required, and biofilm eradication is a critical target for successful management. Topical antibiotic treatments, such as antibiotic-loaded bone cement (ALBC), have been used for some time, and continuous local antibiotic perfusion therapy, a less invasive method, has been developed by our group. However, the optimal antibiotics and concentrations for biofilms of clinical isolates are still not well understood. We examined the efficacy of high concentrations of gentamicin against MRSA biofilms and the role of gentamicin resistance genes in biofilm eradication. We collected 101 MRSA samples from a hospital in Japan and analyzed their gene properties, including methicillin and gentamicin resistance, and their minimum biofilm eradication concentration (MBEC) values. Our results showed that high concentrations of gentamicin are effective against MRSA biofilms and that even concentrations lower than the MBEC value could eliminate biofilms after prolonged exposure. We also identified three aminoglycoside/gentamicin resistance genes [*aac(6′)-aph(2″)*, *aph(3′)-III*, and *ant(4′)-IA*] and found that the presence or absence of these genes may inform the selection of treatments. It was also found that possession of the *aac(6′)-aph(2″*) gene correlated with the minimum inhibitory concentration/MBEC values of gentamicin. Although this study provides insight into the efficacy of gentamicin against MRSA biofilms and the role of gentamicin resistance genes, careful selection of the optimal treatment strategy is needed for clinical application.

**IMPORTANCE:**

Our analysis of 101 MRSA clinical isolates has provided valuable insights that could enhance treatment selection for biofilm infections in orthopedics. We found that high concentrations of gentamicin were effective against MRSA biofilms, and even prolonged exposure to concentrations lower than the minimum biofilm eradication concentration (MBEC) value could eliminate biofilms. The presence of the *aac(6′)-aph(2″)* gene, an aminoglycoside resistance gene, was found to correlate with the minimum inhibitory concentration (MIC) and MBEC values of gentamicin, providing a potential predictive tool for treatment susceptibility. These results suggest that extended high concentrations of local gentamicin treatment could effectively eliminate MRSA biofilms in orthopedic infections. Furthermore, testing for gentamicin MIC or the possession of the *aac(6′)-aph(2″)* gene could help select treatment, including topical gentamicin administration and surgical debridement.

## INTRODUCTION

Bone and soft tissue infections, such as osteomyelitis, fracture-related infections (FRI), and periprosthetic joint infection (PJI), are often difficult to treat with antimicrobial agents. This is because infected foci have poor blood flow, making it difficult for intravenous antimicrobial agents to migrate into the foci (1, 2). Furthermore, bacteria in infected foci tend to form biofilms (3–5). Biofilms are slime-like aggregates surrounded by a polysaccharide matrix secreted by bacteria, and bacteria growing in them cause reduced drug sensitivity, escape from host immunity, and relapse of infection. The minimum inhibitory concentration (MIC) is the concentration required to inhibit the growth of a single free bacterium, but a minimal biofilm eradication concentration (MBEC) of 100 to 1,000 times the MIC is required to eradicate a biofilm (6–8), making it difficult to kill bacteria in biofilms by systemic administration of antimicrobial agents alone.

Local administration of antimicrobials has the advantage of increasing the local concentration of antimicrobials and minimizing their systemic effects. In actual clinical practice, aminoglycoside antimicrobials, including gentamicin, have been used for open fractures (9) and local administration of antimicrobial-containing cement (10, 11), and their efficacy has been confirmed. Continuous local antibiotic perfusion (CLAP) therapy, previously reported by our group (1, 12), provides continuous antibiotic circulation throughout an infected lesion. It is an innovative treatment method that allows local administration of high concentrations of antimicrobials without increasing the daily antimicrobial use by slowing down the flow rate. It is also possible with this method to transfer antimicrobials locally at concentrations effective for the control of biofilm, and it has been reported to be very effective in reducing infection rates in open fractures, infections after bone grafting, peri-implant infections, and deposited pyogenic arthritis (13). Aminoglycoside antibiotics (mainly gentamicin) are also used for CLAP, and high concentrations (1,200 mg/L) of gentamicin are administered to the site of infection to kill antibiotic-resistant biofilm. In addition, by monitoring blood levels, the administered antimicrobial can be maintained at the required concentration for the required time while ensuring safety.

Some bacteria are resistant to aminoglycoside antimicrobials, and the main mechanism of resistance is inactivation by drug modification. Modifying enzymes include (i) aminoglycoside N-acetyltransferase (AAC), (ii) aminoglycoside O-phosphotransferase (APH), and (iii) nucleotide transferase (aminoglycoside O-nucleotidyl transferase, ANT). Among the above three enzymes, mainly (i) and (ii) have been reported to be involved in GM resistance (14).

*Staphylococcus aureus* (*S. aureus*) is the most common pathogen causing bone and soft tissue infections, and it is prone to biofilm formation (15). Among *S. aureus*, methicillin-resistant *Staphylococcus aureus* (MRSA) causes clinical problems because it is often resistant not only to β-lactams but also to many other drugs (16). The resistance of MRSA to aminoglycoside drugs is due to the acquisition of the aforementioned ability to produce drug-modifying enzymes and three types of such genes, *aac(6′)-aph(2*″), *aph(3′) -III*, and *ant(4′) -IA* (17–19).

As noted above, gentamicin is frequently used as a topical agent for bone and soft tissue infections, but it was not originally intended as an antibacterial agent for the treatment of Gram-positive cocci. In cases where MRSA is found to be the causative organism, vancomycin is the first-line antibacterial agent. To make the use of gentamicin for topical administration the standard of care, it is essential to have basic data on the MBEC and the possession of aminoglycoside-modifying enzyme genes, in addition to the MICs of gentamicin against clinical isolates.

In this study, we first examined the MIC and MBEC of gentamicin against 101 MRSA clinical isolates, and clarified their antimicrobial susceptibility pattern and the relationship between MIC and MBEC values. Next, we examined these MRSA strains for the presence of three aminoglycoside resistance genes already known in *S. aureus* [*aac(6′)-aph(2″), aph(3′)-III*, and *ant(4′)-IA*] and analyzed their correlation with the MIC/MBEC values of gentamicin. Finally, to determine the efficacy of a topically administered high-concentration gentamicin against MRSA biofilms, biofilms of MRSA strains were exposed to high concentrations of gentamicin for extended periods of time to determine the bactericidal effect of MRSA in the biofilm.

## RESULTS

### Cefoxitin susceptibility and *mecA* gene carriage of MRSA isolates used in this study

**Fig 1:**
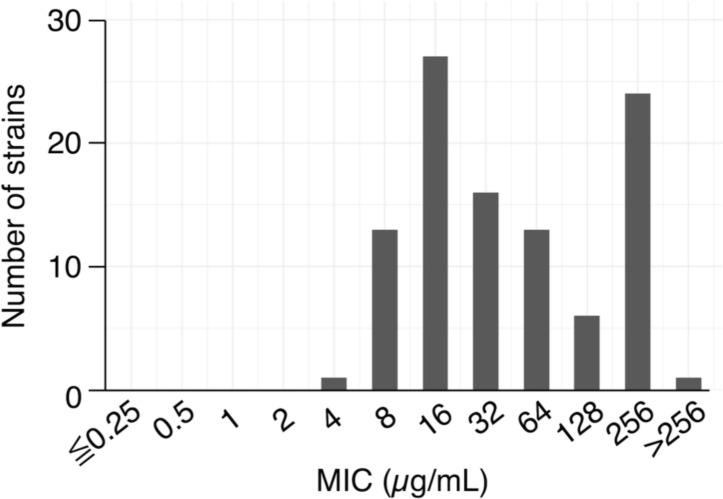
Distribution of MIC values of cefoxitin against clinical isolates of MRSA. Of the 101 isolates, 100 strains had MICs to cefoxitin of 8 µg/mL or higher, confirming that they were MRSA. The remaining one isolate was also confirmed as MRSA with MICs to oxacillin of 4 µg/mL, respectively.

A total of 101 MRSA strains isolated from clinical specimens in the clinical microbiology laboratory of a university hospital and previously identified as resistant to oxacillin or cefoxitin according to CLSI criteria were tested for cefoxitin MIC for confirmation. Of the 101 isolates, 100 strains had MICs to cefoxitin of 8 µg/mL or higher, confirming that they were MRSA (Fig. 1). The remaining one isolate was also confirmed as MRSA with MIC to oxacillin of 4 µg/mL. These 101 MRSA isolates were examined for possession of the *mecA* gene using PCR, and all were found to possess it. Nine representative isolates were analyzed for multi-locus sequence typing, resulting in four distinct sequence types as follows: ST8 (5/9 strains), ST22 (2/9 strains), ST1 (1/9 strains), and ST764 (1/9 strains). This confirmed that our study population consists of strains with diverse genetic backgrounds.

### MICs and MBECs of gentamicin against 101 MRSA clinical isolates

**Fig 2:**
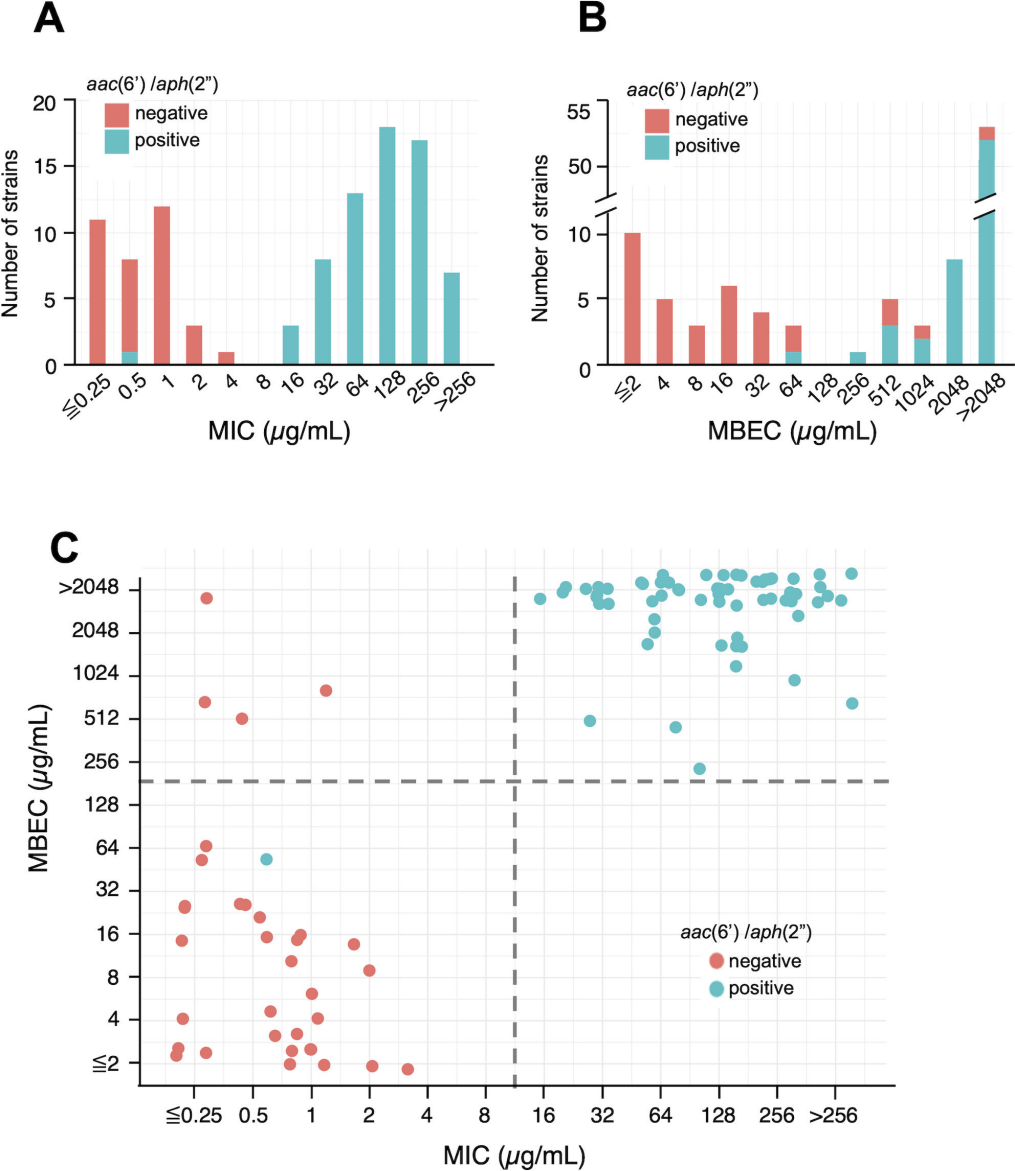
Distribution of MIC and MBEC values of gentamicin against clinical isolates of MRSA. (**A**) Distribution of MIC of gentamicin against the 101 MRSA isolates. There were 66 (65.3%) strains that were resistant to gentamicin (≥ 16 µg/mL). Strains with the *aac(6′)-aph(2″*) gene (67 strains) are shown in blue, and those without the gene (34 strains) are shown in orange. All but one of the strains with *aac(6′)-aph(2*″) were resistant to gentamicin. (**B**) Distribution of MBEC of gentamicin against the 101 MRSA isolates. MBEC values for gentamicin were higher than MIC values and varied between strains: 52 (51.5%) strains had MBEC values above 2,048 µg/mL. Strains with the *aac(6′)-aph(2″*) gene (67 strains) are shown in blue, and those without the gene (34 strains) are shown in orange. (**C**) Relationship between MIC and MBEC values of gentamicin and the presence or absence of the *aac(6′)-aph(2*″) gene. Each dot represents the MIC and MBEC values against each MRSA strain. Strains with the *aac(6′)-aph(2*″) gene (67 strains) are indicated by blue dots and those without the gene (34 strains) by orange dots. The dashed lines indicate the borderline between MIC values of 8 and 16 µg/mL and MBEC values of 128 and 256 µg/mL, respectively.

The MICs of gentamicin for the 101 isolates were determined by the microdilution method (Fig. 2A). There were 66 (65.3%) strains that were resistant to gentamicin (≥ 16 µg/mL). It is noteworthy that each MRSA strain showed a bimodal distribution with each MIC for GM clearly divided into ≤8 and ≥16 µg/mL groups.

Next, the MBEC of gentamicin was determined as the lowest concentration of gentamicin that killed all bacteria in the biofilm in an overnight (approximately 16 h) exposure, reflecting the efficacy of the antibiotics against MRSA biofilms. A modified Calgary Biofilm Device method was used to induce biofilm formation (6). As shown in Fig. 2B, the MBEC values of gentamicin were higher than the MIC values and varied between strains as follows: 32 (31.7%) strains had MBEC values below 128 µg/mL, while the other strains had higher values, especially 52 (51.5%) with MBEC values above 2,048 µg/mL.

The association between gentamicin resistance (MIC ≥16 µg/mL) and MBEC values of ≥256 µg/mL was found to be significant (*P* = 2.2 × 10^−16^, Fisher’s exact test). Out of 35 strains that were susceptible to gentamicin, 31 (88.6%) had MBEC values below 128 µg/mL, while all resistant strains showed MBEC values of 256 µg/mL or more.

### Relationship between aminoglycoside resistance gene carriage and MIC/MBEC of gentamicin in 101 MRSA clinical isolates

We examined 101 MRSA isolates using PCR to determine the relationship between aminoglycoside resistance and the presence of three aminoglycoside-modifying enzyme (AME) genes in MRSA clinical isolates. The results showed that 67 (66.3%) of the isolates were found to carry the *aac(6′)-aph(2″*) gene. Fifteen strains (14.9%) were found to carry the *ant(4′)-Ia* gene, of which 14 strains were accompanied by the *aac(6′)-aph(2″*) gene. No strains carried the *aph(3′)-III* gene.

All but one of the strains carrying the *aac(6′)-aph(2″*) gene were resistant to gentamicin, with an MIC_50_ of 128 µg/mL and an MIC_90_ of 256 µg/mL. In contrast, all of the strains not carrying this gene were susceptible to gentamicin, with an MIC_50_ of 0.5 µg/mL and an MIC_90_ of 2 µg/mL (Fig. 2A). These findings suggested a strong correlation between the *aac(6′)-aph(2″*) gene and gentamicin resistance (MIC ≥16 µg/mL) among the 101 MRSA isolates, which was statistically significant (*P* < 2.2 × 10^−16^, Fisher’s exact test). Similarly, strains carrying the *aac(6′)-aph(2″*) gene tended to show MBEC values of 256 µg/mL or higher to gentamicin, with an MBEC_50_ that was >2,048 µg/mL. In contrast, strains without the gene had an MBEC_50_ of 8 µg/mL and an MBEC_90_ of 512 µg/mL (Fig. 2B). Statistically, the presence of the *aac(6′)-aph(2″*) gene in MRSA isolates was significantly associated with MBEC values of ≥256 µg/mL (*P* < 2.2 × 10^−16^, Fisher’s exact test).

On the other hand, the strains with the *aac(6′)-aph(2″*) gene that additionally carried the *ant(4′)-Ia* gene showed no statistically significant difference in MBEC values of ≥256 µg/mL and ≥2,048 µg/mL (*P* = 1.000 and 0.3301, respectively, Fisher’s exact test). Among 53 strains with the *aac(6′)-aph(2″*) gene but without the *ant(4′)-Ia* gene, 52 (98.1%) had MBEC values of ≥256 µg/mL, and 46 (86.8%) had MBEC values of ≥2,048 µg/mL. For all 14 strains with both the *aac(6′)-aph(2″*) gene and the *ant(4′)-Ia* gene, the MBEC values were 2,048 µg/mL or more. Therefore, we cannot conclude that having both the *aac(6′)-aph(2″*) and *ant(4′)-Ia* genes confers greater resistance than having only one of those genes.

To clarify the relationship between the *aac(6′)-aph(2″*) gene carriage and MIC/MBEC values, all strains were color coded according to the presence or absence of the *aac(6′)-aph(2″*) gene, and each was plotted against MIC and MBEC values, as shown in Fig. 2C. As mentioned above, gentamicin resistance (MIC ≥16 µg/mL) in the isolates was significantly associated with MBEC values ≥ 256 µg/mL. The strains with the *aac(6′)-aph(2″*) gene showed a predominant distribution in the high MIC (≥ 16 µg/mL) and high MBEC (≥ 256 µg/mL) range, with only one exception.

One strain (CM19) tested positive for the presence of the *aac(6′)-aph(2″*) gene through PCR, but it was still susceptible to gentamicin. We have sequenced the *aac(6′)-aph(2″*) gene of CM19 and three other randomly selected strains resistant to gentamicin. Our analysis revealed that CM19 had an insertion of IS256 in the *aac(6′)-aph(2″*) gene, whereas the gene sequences of the other three strains were identical and not mutated.

### Relationship between biofilm formation capacity and MBEC of gentamicin in 101 MRSA clinical isolates

The matrix of the biofilm may prevent the penetration of gentamicin, which is considered to contribute to MRSA resistance in the biofilm. To confirm this, we measured the amount of matrix production of each isolate by Crystal Violet staining, but no difference in the amount of matrix production was found between the groups with MBEC values below 128 and above 256 mg/dL (mean OD_570_ ±standard error; MBEC ≤ 128 vs ≥256 mg/dL; 0.0175 ± 0.00191 vs 0.0180 ± 0.00215, *P* = 0.872). This result suggests that the *aac(6′)-aph(2″)* gene is a key factor in MRSA biofilm resistance to gentamicin.

### Bactericidal effect of prolonged exposure to high concentrations of gentamicin on MRSA biofilms showing high MBEC values

**Fig 3:**
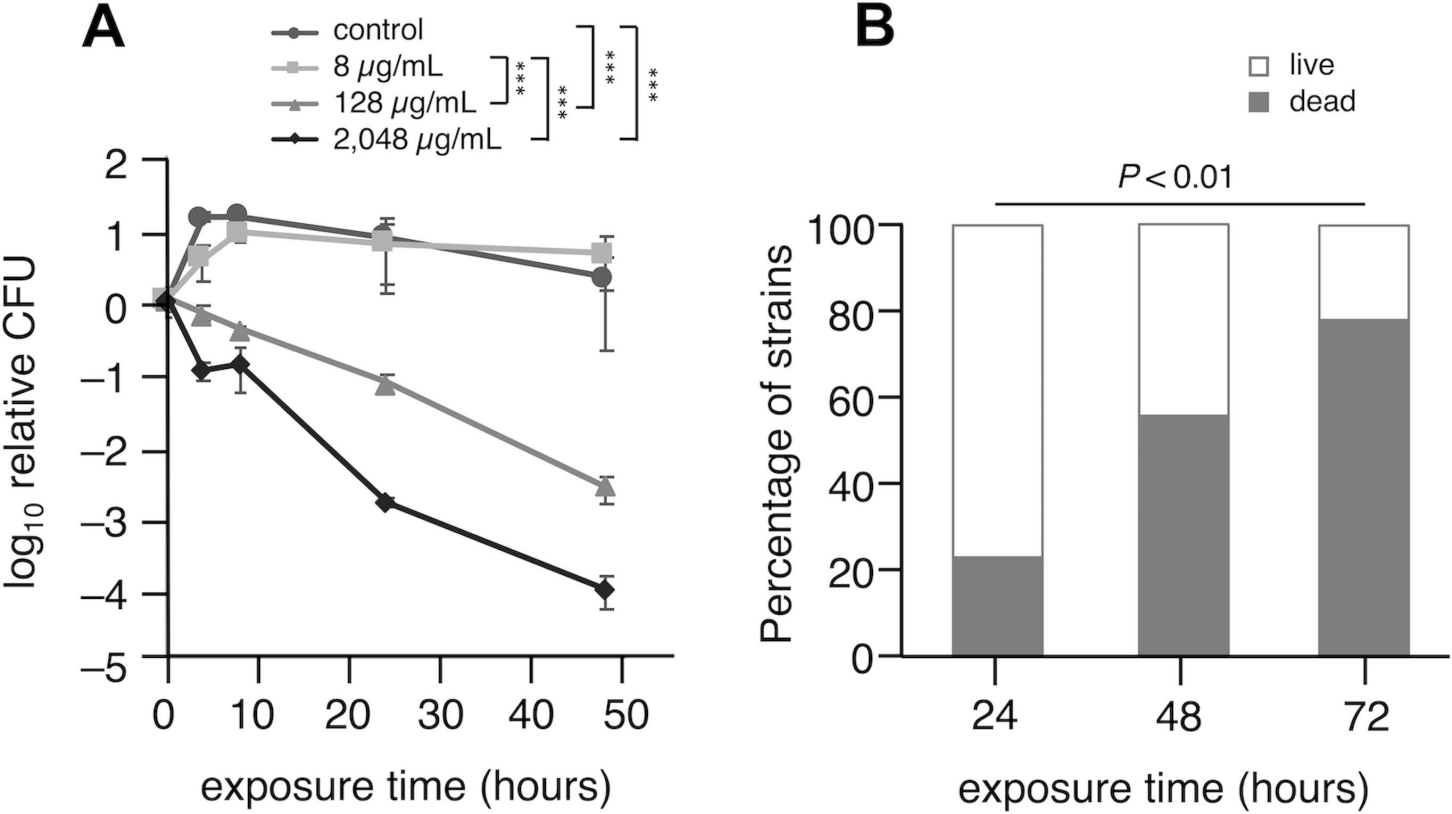
Effect of prolonged gentamicin exposure on biofilms of MRSA clinical isolates. (**A**) Time–killing curve of MRSA biofilm after prolonged gentamicin exposure. Biofilms of one randomly selected clinical isolate of MRSA, carrying the *aac(6′)-aph(2″)* gene and an MBEC greater than 2,048 µg/mL, were exposed to gentamicin at concentrations of 0, 8, 128, and 2,048 µg/mL for 4, 8, 24, and 48 h to examine its bactericidal effect over time. The number of CFUs in the biofilm before exposure to gentamicin was set as 1, and the number of CFUs at each time point was expressed as a logarithm. The analysis was performed in triplicate. Two-way ANOVA with Bonferroni *post hoc* test was used to examine the statistical significance between groups. ***P < 0.001. (**B**) Percentage of MRSA biofilm eradicated by prolonged gentamicin exposure. A total of 51 strains with the *aac(6′)-aph(2″)* gene and MBEC values greater than 2,048 µg/mL were tested. All biofilms of all strains were exposed to gentamicin at a concentration of 1,024 µg/mL for 24, 48, and 72 h to determine the percentage of strains with viable bacteria in the biofilm. The percentage of strains surviving in the biofilm is shown in white, and the percentage of dead strains is shown in gray. Chi-squared test was used to examine the statistical significance between groups.

We randomly selected one clinical isolate of MRSA that carried the *aac(6′)-aph(2″)* gene and had an MBEC of >2,048 µg/mL, and exposed the biofilm of this isolate to various concentrations of gentamicin for up to 48 h to determine its bactericidal effect over time. As shown in Fig. 3A, this strain showed a significant decrease in the number of CFU after 24 h of exposure at concentrations above 128 µg/mL and a 3-log decrease in biofilm CFU after 48 h at concentrations of 2,048 µg/mL. Notably, these results suggest that prolonged exposure to high concentrations of gentamicin can kill bacteria in biofilms, even if the concentration is below the MBEC of the strain.

Based on these results, biofilms of 51 strains carrying the *aac(6′)-aph(2″)* gene and with MBEC values greater than 2,048 µg/mL were exposed to gentamicin at a concentration of 1,024 µg/mL for up to 72 h to examine bacterial viability in the biofilm. As shown in Fig. 3B, the percentages of the strains with detectable survival in biofilm decreased significantly over time: from 77.5% at 24 h, to 45.1% at 48 h, and further to 22.5% at 72 h (*P* < 0.01, χ^2^ test). These data suggest that a high concentration and long duration of gentamicin exposure have the potential to eliminate MRSA biofilms, even in strains carrying the *aac(6′)-aph(2″)* gene and with MBEC values >2,048 µg/mL.

## DISCUSSION

Aminoglycoside antimicrobials, with the exception of arbekacin (ABK), are not usually used in the treatment of MRSA infections by intravenous administration. Therefore, few reports have focused on the MICs and MBECs of aminoglycosides against MRSA clinical isolates (20–25). However, since gentamicin, an aminoglycoside antimicrobial agent, is frequently used as a topical antimicrobial agent for bone and soft tissue infections (9, 10, 26, 27), it is important to study the MIC and MBEC of gentamicin against MRSA clinical isolates. In this study, we examined the MICs and MBECs of gentamicin against 101 MRSA isolates from one hospital in 2020 and obtained the following two main findings:

1. Of the MRSA clinical isolates, 65.3% were resistant (≥16 µg/mL) to gentamicin. The isolates were clearly divided into two groups as follows: strains with low MIC (≤8 µg/mL) of gentamicin (susceptible strains) and strains with high MIC (≥16 µg/mL) of gentamicin (resistant strains) (Fig. 2A).
2. The MBEC was higher than the MIC, and 51.5% of the isolates showed an MBEC >2,048 µg/mL (Fig. 2B). Isolates with a higher MIC also had significantly higher MBEC (Fig. 2C).

These results suggest that, when gentamicin is used for local treatment of MRSA infections, it should be noted that many strains have a high MBEC.

In theory, biofilms can be eradicated if the organism can be exposed to high concentrations of antibiotic solutions (20, 25, 28). This strategy is used to treat biofilm-associated infections, such as antibiotic lock therapy (29) and, more recently, CLAP therapy, which we developed (1, 12, 13). We exposed MRSA biofilms to gentamicin at a concentration of 1,200 µg/mL, which is actually used in current CLAP treatment, and examined the survival rate of bacteria in the biofilm over time. In this experiment, MBEC values were defined as the minimum biofilm eradication concentration for overnight (approximately 16 h) antimicrobial exposure, although they were previously reported to decrease with increasing antimicrobial exposure time (28). The results showed that even for biofilms of MRSA clinical isolates with MBEC of >2,048 µg/mL of gentamicin, exposure to gentamicin at a concentration of 1,200 µg/mL for 48 h killed approximately 60% of the strains, and after 72 h, approximately 80% were killed (Fig. 3B). Exposure of the biofilm to gentamicin at a concentration of 128 µg/mL also killed the MRSA in the biofilm over time (Fig. 3A). This suggests that even if the concentration of gentamicin reaching the site of MRSA infection is lower than the MBEC value, the biofilm can be eradicated by exposure for a long period of time. It can be assumed that CLAP therapy can adequately control MRSA that have formed biofilms, since it injects a high concentration of gentamicin locally and perfuses the area over a long period of time.

Small colony variants of *S. aureus* (SA-SCVs) are known to play a role in the failure of antibiotic treatment and the recurrence of infections in orthopedics (30, 31). This is due to an increase in biofilm formation, changes in susceptibility to antibiotics, and the ability to persist inside cells, evading the host immune response. Dysfunction in metabolic pathways, such as the electron transport chain or tricarboxylic acid cycle, results in decreased electron transport and impairs aminoglycoside uptake (32). The MIC of gentamicin against the SA-SCV was reported to be up to 32 times higher than that of wild-type strains (33). Furthermore, treatment with aminoglycosides can facilitate the emergence of SA-SCVs (34). SA-SCVs may be present in our *in vitro* model, and our findings indicate that they could be eliminated by prolonged exposure to a high concentration of gentamicin. However, since entering host cells might decrease their susceptibility to antimicrobials (33), we need to further study the effectiveness of high-concentration antimicrobial therapy against SA-SCVs in the presence of host cells.

It has been reported that the *aac(6′)-aph(2″)* gene, one of the three aminoglycoside resistance genes known in *S. aureus*, is widely distributed in clinical isolates of MRSA (35). In this study, we found that most of the MRSA clinical isolates with high MIC (≥16 µg/mL) and high MBEC (≥256 µg/mL) harbored the *aac(6′)-aph(2″)* gene. The enzyme encoded by the *aac(6′)-aph(2″)* gene is known to be a two-headed enzyme with two simultaneous functions: aminoglycoside N-acetyltransferase (AAC) and aminoglycoside O-phosphotransferase (APH) (35, 36). Fifteen MRSA isolates were found to carry the *ant(4′)-Ia* gene, 14 of which also carried the *aac(6′)-aph(2″)* gene simultaneously, but there was no evidence that aminoglycoside resistance was further enhanced by the presence of these two genes. Based on these results, if MRSA positive for the *aac(6′)-aph(2″)* gene is isolated from clinical specimens, its MIC/MBEC value is expected to be high. If the expected therapeutic effect is not observed, methods other than topical gentamicin administration, such as surgical resection, may be required.

The insertion of IS256 into the *aac(6′)-aph(2″)* gene was considered to prevent the expression of the aminoglycoside-modifying enzyme, making the strain more susceptible to gentamicin. Kime and colleagues recently reported a similar phenomenon (37). Interestingly, they observed that the mutated gene reverted to its unmutated sequence and regained function with high frequency after exposure to gentamicin. This suggests that high concentrations of gentamicin may be required when the *aac(6′)-aph(2″)* gene is present, whether mutated or not.

There are some limitations in this study. First, all of the MRSA strains analyzed in this study were isolated from a single institution, and patient information was unknown. Future studies may need to be conducted at multiple institutions and with strains derived from bone and soft tissue infections. Second, gentamicin was the only antimicrobial agent examined in this study. Theoretically, even higher concentrations of topically administered gentamicin would be expected to be more effective in eradicating MRSA biofilms. It has been reported, however, that high blood concentrations of antimicrobials can cause systemic side effects, and very high concentrations of antimicrobials inhibit osteoblast replication and cause cell death (38, 39). In our clinical setting, we usually measure the blood gentamicin concentration to ensure safety and maintain it below 1 µg/mL. Given that no method has yet been established to accurately measure local antimicrobial concentrations, such as those in intra-articular or intramuscular sites, further studies are needed to elucidate the relationship between local concentration and efficacy. In addition, future studies should examine the efficacy of other aminoglycoside antimicrobial agents in the removal of biofilms or the efficacy of gentamicin in combination with other antimicrobial agents. Finally, this study is an observational study. This study represents a critical step in understanding the factors that predict MBEC levels of isolates in hospital settings. However, we acknowledge that our results only demonstrated a correlation and that further experiments using mutant strains are necessary to establish a causal relationship.

In summary, we analyzed the MIC and MBEC of gentamicin against MRSA clinical isolates and found that high concentrations of gentamicin were effective in eliminating MRSA biofilms, and that prolonged exposure to gentamicin at concentrations lower than the MBEC value can eliminate biofilms. Furthermore, among the aminoglycoside resistance genes known in MRSA, the *aac(6′)-aph(2″)* gene correlates with MIC/MBEC values, and testing for its presence in patient isolates may be useful in selecting treatment strategies.

## MATERIAL AND METHODS

### Bacterial strains

A total of 101 MRSA strains isolated from clinical specimens at the University Hospital of Occupational and Environmental Health in 2020 and identified as oxacillin- or cefoxitin-resistant according to CLSI criteria by the hospital’s clinical laboratory were used in this study. The screening and identification of MRSA were carried out in the hospital’s clinical laboratory. Specifically, all isolates were identified at the species level using the MALDI Biotyper (Bruker Daltonics, Bremen, Germany). The isolates identified as *S. aureus* were then tested for susceptibility to cefoxitin and oxacillin following the CLSI M100-S22 guidelines (40). An isolate was classified as MRSA if it showed resistance to either cefoxitin or oxacillin. The study analyzed only one isolate per patient. When multiple MRSA strains were obtained from a single patient, we chose the earliest one and excluded the others. To clarify the diversity of isolates we used, we randomly selected nine strains and performed multi-locus sequence typing (MLST) as described previously (41). The allelic profiles and sequence types (STs) were assigned using the MLST website (42). All strains were stored in brain heart infusion broth (BHI) with 20% glycerol and maintained at −80°C until used for analysis.

The bacteria used in this study were isolated for the purpose of diagnosing and treating patients. No patient information was collected for the study. Therefore, according to the Ethical Guidelines for Medical and Health Research Involving Human Subjects issued by the Ministry of Health, Labour and Welfare of Japan, this study was not subject to ethical review.

### MIC assays

Stored MRSA strains were cultured on Nutrient Agar plates and suspended in phosphate-buffered saline (PBS) to a concentration of 10^6^ CFU/mL. Cefoxitin and gentamicin were purchased from Sigma-Aldrich, St. Louis, MO. The antimicrobial solutions were prepared using cation-adjusted Müller–Hinton broth (CAMHB, Sigma-Aldrich, St. Louis, MO) in accordance with Clinical and Laboratory Standards Institute guidelines.

Serial dilutions (0.25–256 µg/mL) of each antibacterial agent were mixed with an equal volume of bacterial suspensions on 96-well microplates and incubated overnight at 37°C. The lowest concentration that inhibited visible bacterial growth was defined as the MIC.

### MBEC assays

In this study, an MRSA biofilm was defined as a structure in which the bacteria adhere so strongly to the plastic surface that repeated washings do not dislodge them, and Crystal Violet staining shows matrix production (43, 44). MRSA strains were suspended in BHI broth supplemented with 2% glucose and 2% sucrose (45) to a concentration of 10^5^ CFU/mL. To form a biofilm, a lid with 96 pegs (Nunc Immuno TSP Lids, Thermo Scientific) was immersed in the suspension and incubated at 37 ˚C overnight (approximately 16 h). Initially, the Calgary Biofilm Pin Lid Device method of biofilm fabrication involved culture with sheer stress caused by a rocking motion (6). However, as a preliminary examination, we cultured biofilms using shaking and stationary methods under the same conditions and found no difference in measurements at OD 620 after staining with Crystal Violet. Therefore, we proceeded with preparing biofilms using static culture.

After the growth of biofilm, the lid was washed twice with PBS and immersed in the antimicrobials (2–2,048 µg/mL) prepared in a 96-well plate. After incubation at 37 ˚C for 24 h, the lid was washed twice with PBS and was sonicated for 10 min in 180 µL of CAMHB to detach the residual biofilms from the pegs. The detached biofilm was collected and spotted on a nutrient agar plate. After incubation at 37 ˚C for 16 to 24 h, the number of colonies was counted. The lowest concentration that exhibited a colony count below 0.1% of the control was designated as the MBEC.

All experiments were conducted in triplicate. At least two of the triplicate measurements had to match for a result to be considered valid. When the results from the triplicate did not agree, the experiment was repeated seven times to ensure reliability and consistency.

### Detection of *mecA* gene and aminoglycoside resistance gene using PCR

Bacterial DNA was extracted from the culture of each MRSA strain using the illustra bacteria genomicPrep Mini Spin kit (GE Healthcare, Buckinghamshire, United Kingdom) according to the protocol specified for Gram-positive bacteria.

The *mecA* gene PCR was performed using primers mecA-F (5′-GTAGAAATGACTGAACGTCCGATAA-3′) and mecA-R (5′-CCAATTCCACATTGTTTCGGTCTAA-3′), Ex Taq buffer (TaKaRa, Otsu, Shiga, Japan), deoxynucleoside triphosphate, extracted DNA, and Ex Taq HS DNA polymerase (TaKaRa, Otsu, Shiga, Japan). The DNA of each MRSA of the isolates was amplified in a thermal cycler under the following conditions: initial denaturation at 95°C for 5 min, 30 cycles of 95°C for 1 min, 57°C for 1 min, 72°C for 1 min, and final extension at 72°C for 10 min.

Aminoglycoside resistance genes of *aac(6′)-aph(2″)*, *ant(4′) -Ia*, and *aph(3′)-III* were detected using PCR, as previously reported (14). The following primer sets were used for the PCR of each gene: aacA-aphD_F (5′-CCAAGAGCAATAAGGGCATACC-3′) and aacA-aphD_R (5′-CACACTATCATAACCACTACCG-3′) for the *aac(6′)-aph(2″)* gene, aadD_F (5′-AATCGGTAGAAGCCCAA-3′) and aadD_R (5′-GCACCTGCCATTGCTA-3′) for the *ant(4′)-Ia* gene, and aphA_F (5′-CTGATCGAAAAATACCGCTGC-3′) and aphA_R (5′-TCATACTCTTCCGAGCAAAGG-3′) for the *aph(3′)-III* gene, respectively. Amplification of each gene was performed under the following conditions: initial denaturation at 95°C for 5 min; followed by 30 cycles of denaturation at 95°C for 1 min, annealing for 1 min at 45°C for the *aac(6′)-aph(2″)* gene*,* 47°C for the *ant(4′)-Ia* gene, 55°C for the *aph(3′)-III* gene, and extension at 72°C for 1 min; and final extension at 72°C for 10 min. DNA amplification was confirmed using gel (1.5%) electrophoresis at 100 V.

For sequence analysis of the *aac(6′)-aph(2″)* gene, extracted DNA was subjected to PCR targeting the whole length of the gene. The cycling condition was 95°C for 5 min, followed by 30 cycles of 95°C for 1 min, 52°C for 1 min, and 72°C for 3 min. The PCR products underwent DNA sequence analyses (Fasmac, Atsugi, Japan). Primers for PCR and DNA sequencing are listed in Table S1. All the sequences were submitted to a public database (DNA data bank of Japan, Accession number: LC830472-LC830475).

### Measurement of biofilm matrix in MRSA clinical isolates

The amount of matrix production of each isolate was measured by Crystal Violet staining. Peg lids with biofilm to be measured were removed from the culture medium, washed twice with PBS for 1 min each, and stained with 0.5% Crystal Violet solution for 10 min. The stained peg lids were washed twice with PBS for 1 min each, and then immersed in 99.5% ethanol to elute the Crystal Violet. The absorbance of the eluate was measured at 570 nm, with the peg that was not inoculated with bacteria as the blank.

### Bactericidal effect of long-term exposure to gentamicin on MRSA strains in biofilms

Biofilms of MRSA clinical isolates carrying the *aac(6′)-aph(2″)* gene and with MBEC >2,048 µg/mL were prepared as described above using 96 peg lids.

For one isolate, biofilms were exposed to 0, 8, 128, and 2,048 µg/mL of gentamicin for 4, 8, 24, and 48 h. After the end of exposure, the lids were washed twice with PBS and sonicated for 10 min to detach the biofilm from the pegs. The detached biofilms were collected and plated on nutrient agar medium with a step dilution in PBS, and CFU was calculated after 24 h of incubation at 37°C. The analysis was performed in triplicate.

On the other hand, the biofilms of all of the strains were exposed to 1,024 µg/mL of gentamicin for 24, 48, and 72 h. The solution of gentamicin was replaced every 24 h. After the exposure, the lids were washed twice with PBS and sonicated in 180 µL of CAMHB for 10 min to detach the remaining biofilm from the pegs. The broth containing the detached biofilm was incubated at 37°C for 24 h, and the survival of MRSA bacteria in the biofilms was determined by observing the growth of the bacteria by the turbidity of the medium. Two sets of biofilms were prepared for each time point for accuracy.

### Statistical analysis

Fisher’s exact test was used to compare the MBEC in the different groups. The amount of matrix production between the two groups was compared by Student *t*-test. A comparison of CFU between multiple groups was performed using the two-way analysis of variance following pairwise *post hoc* comparisons with Bonferroni correction. Chi-squared test was used to analyze the differences in MRSA survival in biofilms over time. Statistical analyses were performed using R (ver. 4.3.1), and *P* < 0.05 was considered significant.

## Data Availability

The aac(6′)-aph(2″) gene sequences of MRSA clinical isolates have been deposited in DDBJ/EMBL/GenBank (accession no. LC830472-LC830475).

## SUPPLEMENTAL MATERIAL

Table S1 spectrum.00647-24-s0001.docx List of primers used for sequencing *aac(6′)-aph(2″)* gene.

ASM does not own the copyrights to Supplemental Material that may be linked to, or accessed through, an article. The authors have granted ASM a non-exclusive, world-wide license to publish the Supplemental Material files. Please contact the corresponding author directly for reuse.
