# Supplementary material for: Effective biofilm eradication in MRSA isolates with aminoglycoside-modifying enzyme genes using high-concentration and prolonged gentamicin treatment
Source: Microbiol Spectr. 2024 Aug 27;12(10):e00647-24. doi: 10.1128/spectrum.00647-24 (PMC11448082; doi:10.1128/spectrum.00647-24)
Supplement: Table S1 — List of primers used for sequencing aac(6′)-aph(2″) gene. [file spectrum.00647-24-s0001.docx]

**Table S1**

List of primers used for sequencing *aac(6')-aph(2'')* gene

| Primer name | Prime sequence (5' → 3') |
| --- | --- |
| aac-aph_seqF1 | TAGAGCTTGCCATGTATATG |
| aac-aph_seqF2 | CAAGAGCAATAAGGGCATAC |
| aac-aph_seqF3 | GATATTAGTGAATGTACTATTG |
| aac-aph_seqF4 | CTGAAAAGCGAAGAGATTC |
| aac-aph_seqF5 | CGGTTTACAAGGTACGG |
| aac-aph_seqR1 | ATGTCTTTTATAATAGCGTTTC |
| aac-aph_seqR2 | CAATAGTACATTCACTAATATC |
| aac-aph_seqR3 | GTATGCCCTTATTGCTCTTG |
| aac-aph_seqR4 | CCGTACCTTGTAAACCG |
| aac-aph_seqR5 | GAATCTCTTCGCTTTTCAG |
